# Clinical approaches for poststroke seizure: a review

**DOI:** 10.3389/fneur.2024.1337960

**Published:** 2024-04-10

**Authors:** Han Uk Ryu, Hong Jin Kim, Byoung-Soo Shin, Hyun Goo Kang

**Affiliations:** ^1^Department of Neurology, Jeonbuk National University Medical School and Hospital, Jeonju, Republic of Korea; ^2^Research Institute of Clinical Medicine of Jeonbuk National University – Biomedical Research Institute of Jeonbuk National University Hospital, Jeonju, Republic of Korea

**Keywords:** poststroke seizure, unilateral weakness, dysarthria, sensory deficit, stroke

## Abstract

Poststroke seizure is a potential complication of stroke, which is the most frequent acute symptomatic seizure in adults. Patients with stroke may present with an abnormal or aggressive behavior accompanied by altered mental status and symptoms, such as hemiparesis, dysarthria, and sensory deficits. Although stroke manifestations that mimic seizures are rare, diagnosing poststroke seizures can be challenging when accompanied with negative postictal symptoms. Differential diagnoses of poststroke seizures include movement disorders, syncope, and functional (nonepileptic) seizures, which may present with symptoms similar to seizures. Furthermore, it is important to determine whether poststroke seizures occur early or late. Seizures occurring within and after 7 d of stroke onset were classified as early and late seizures, respectively. Early seizures have the same clinical course as acute symptomatic seizures; they rarely recur or require long-term antiseizure medication. Conversely, late seizures are associated with a risk of recurrence similar to that of unprovoked seizures in a patient with a focal lesion, thereby requiring long-term administration of antiseizure medication. After diagnosis, concerns regarding treatment strategies, treatment duration, and administration of primary and secondary prophylaxis often arise. Antiseizure medication decisions for the initiation of short-term primary and long-term secondary seizure prophylaxis should be considered for patients with stroke. Antiseizure drugs such as lamotrigine, carbamazepine, lacosamide, levetiracetam, phenytoin, and valproate may be administered. Poststroke seizures should be diagnosed systematically through history with differential diagnosis; in addition, classifying them as early or late seizures can help to determine treatment strategies.

## 1 Introduction

The incidence of stroke has increased by 50% over the past two decades, with a 70% increased incidence from 1990 to 2019 (1). Poststroke seizures are important complications of stroke, as the risks of mortality and morbidity are higher in stroke patients who experience seizure than in those who do not (2). In the past, poststroke seizures accounted for approximately 16% of all seizures; however, with the increasing incidence of stroke, a recent study indicated that 73% of acute symptomatic seizures in individuals >18 years of age are associated with stroke (3, 4). Late poststroke seizures tend to progress to poststroke epilepsy, with a recurrence rate of 71.5% within 10 years, necessitating the administration of antiseizure medications (5). The incidence of poststroke epilepsy was 6.4 and 12.4% in ischemic and hemorrhagic strokes, respectively, based on the analysis of over 100,000 stroke cases in the United Kingdom and Sweden (6, 7). However, the predictive factors for poststroke seizures are not well-established, and elucidating them is difficult owing to other causes of seizures besides stroke.

Differential diagnosis is crucial because patients with stroke often have comorbidities and may present with symptoms similar to poststroke seizures. This could be attributed to the aggressive or abnormal behavior observed in patients with stroke, accompanied with altered mental status and delirious conditions, abnormal movement disorders, and autonomic dysfunction, as well as relatively well-known neurological deficits, such as unilateral weakness, dysarthria, and sensory deficits, which depend on disease severity and stroke location. Epileptic seizures accompanied with negative postictal symptoms may mimic stroke, making diagnosis difficult (3). After diagnosis of poststroke seizures, concerns exist regarding the type of antiseizure medication (ASM) to be administered, duration of administration, and use of ASM to prevent recurrent seizures.

Stroke incidence has risen over the last few decades, and with post-stroke seizures being a complication with impacts on morbidity, mortality, and treatment, an understanding of the definition, diagnostic evaluation, treatment, and future study directions of post-stroke seizures and epilepsy is warranted.

## 2 Definition

### 2.1 Acute symptomatic (early) vs. unprovoked (late) seizures

Acute symptomatic seizures due to stroke are closely related to the location and severity of brain damage; therefore, a causal relationship should be inferred if the brain lesion causing the seizures is clearly identified and the seizure occurs with close temporal continuity (8). Some previous studies set 7 d after stroke as the threshold for distinguishing between early and late seizures, whereas others determined 14 d after stroke as the threshold (Table 1). The International League against Epilepsy (ILAE) classifies poststroke seizures into early and late seizures using a 7-d threshold; under this classification, seizures occurring within and after 7 d of stroke onset are classified as early and late seizures, respectively (Table 1) (16). Accordingly, the incidences of early and late seizures were 3–6 and 12%, respectively (9, 17, 18). Early seizures are characterized by increased inflammatory responses, changes in neuronal signaling related to protein synthesis, and increased excitatory neurotransmitter (glutamate) release, ionic imbalance, blood–brain barrier (BBB) permeability, breakdown of membrane phospholipids, release of free fatty acids, and oxidative stress (19, 20). Therefore, metabolic disturbances, such as electrolyte imbalances, acid–base disturbances, and glucose instability, may develop; however, most cases are transient and reversible (21). Late seizures are characterized by irreversible changes, such as gliosis, selective neuronal loss, chronic inflammation, angiogenesis, neurodegeneration, collateral synaptic sprouting, and synaptic plasticity (19). Thus, early seizures follow the course of acute symptomatic seizures, whereas late seizures follow the course of unprovoked seizures (5, 22, 23). Moreover, mortality and disability rates were higher in late seizures than in early seizures. In clinical practice, an evidence-based approach showed that the criteria for a situation that could stipulate the seizure recurrence risk as ≥60% (24). Classifying poststroke seizures into the early and late types is useful for distinguishing acute symptomatic and unprovoked seizures. According to the European guidelines, a poststroke seizure occurring after 1 week on stroke onset is considered a late seizure (i.e., an unprovoked seizure) (25).

**Table 1:** Incidence of poststroke epilepsy in early and late seizures in stroke patients.

					Outcome					
Authors	Study type	Enrolled patients	Number of patients	Patient age (years)	ES cases	LS cases	Poststroke epilepsy cases after ES	Poststroke epilepsy cases after LS	Poststroke epilepsy cases at the end of follow-up	Mean follow-up period
Lamy et al. (9)	Prospective & Retrospective	Ischemic stroke	581	42.5 ± 9.0	14 (≤1 week)	20 (<1 week)	6 (LS)/14 (42.8%)	11/20 (55%)	11	3.8 ± 9.7 months
Arntz et al. (10)	Prospective	Stroke	697	40.5 ± 7.8	25 (≤1 week)	53 (>1 week)	8/25 (32%)	31/54 (57.4%)	39	9.1 ± 8.2 years
Naess et al. (11)	Retrospective	Ischemic stroke	232	15–44	4 (≤1 week)	20 (>1 week)	N/A	N/A	N/A	5.7 years
Bladin et al. (12)	Prospective	Stroke	1987	72 ± 11.5	99 (≤2 weeks)	69 (>2 weeks)	6/99 (6%)	41/69 (59%)	47	9 months
Olafsson et al. (13)	Population based	SAH by RCA	44	18–53	10 (≤2 weeks)	N/A	7/10 (70%)	N/A	11	23.1 years (2–37)
Sung et al. (14)	Retrospective	ICH	1,402	11–90	38 (≤2 weeks)	26 (>2 weeks)	11/38 (28.9%)	24/26 (92.3%)	35	20 months (ES) 22 months (LS)
Qian et al. (15)	Population based	ICH	935	69 ± 12	72* (≤2 weeks)	58 (>2 weeks)	19/72 (26.3%)	49/58 (84.4%)	68	2.7 years

SAH, subarachnoid hemorrhage; RCA, ruptured cerebral aneurysm; ICH, intracerebral hemorrhage; ES, early seizure; LS, late seizure; N/A, not available.

*51 cases of immediate seizures (<24 h after ICH) and 21 cases of early seizures (24 h to 2 weeks after ICH).

## 3 Predictors of poststroke seizures

Stroke is a common cause of epileptic seizures in older adults (19). However, there is no international consensus on a poststroke seizure risk prediction model. Systematic reviews and meta-analyses on comparative tests of poststroke seizure multivariate risk prediction models were limited by the potential risk for bias and the clinical heterogeneity of patients (26). Several factors may be useful to establish predictors by considering the risk factors for seizures and the poststroke condition.

### 3.1 Structural etiologies due to stroke

The prevalence of acute symptomatic seizures with intracranial hemorrhage is higher than that of ischemic stroke (10–16% vs. 2–4%) (27, 28). Ischemic strokes with hemorrhagic transformation have a higher seizure risk compared to ischemic strokes alone (29).

There is an increased incidence of poststroke seizures in cases with cortical involvement, total anterior circulation infarction, severe stroke with larger lesions, and functional deficits (25, 30). Reperfusion injury can manifest as blood brain barrier disruption, cortical irritation, and epileptic seizures (31). In late poststroke seizures, upregulation in endostatin and NCAM, and downregulation in S100B, Hsc70, and TNF-R1 in acute phase blood samples of stroke showed a high correlation (32). However the seizure risk for cerebral venous thrombosis is debatable, as some studies suggest an increase of up to 34%, whereas other studies indicate no association (33–35).

### 3.2 Seizure etiologies other than stroke

Acute symptomatic or provoked seizures can be induced by direct and immediate causes (27), including metabolic conditions, central nervous system (CNS) infections, sepsis, trauma, drugs, and alcohol consumption (36).

Determining the causes of acute symptomatic seizures other than stroke lesions is important. Acute symptomatic seizures due to metabolic disturbances are associated with the metabolic condition’s rate of deterioration; the faster the deterioration rate, the higher the risk of seizure (37). Metabolic conditions are usually determined by electrolyte tests conducted within 24 h of seizure. However, no absolute cutoff values for seizure prediction have been established, and only few studies have proposed cutoff values as references (Table 2) (27, 36, 37). Seizures in metabolic disturbances with an electrolyte value below the cutoff should be classified into an unknown category and follow-up should be conducted (8). Acute symptomatic seizures due to CNS infection can be considered acute symptomatic seizures even after 7 d, depending on the clinical course or laboratory findings, as the criteria remain unclear (8). Sepsis can induce encephalopathy, which leads to convulsive or nonconvulsive seizures by triggering the electric circuits that promote seizures (42). Alcohol-withdrawal acute symptomatic seizures should be considered in a patient with a history of excessive alcohol use who develops generalized tonic–clonic seizures after alcohol abstinence for 7–48 h (8). As alcohol-induced acute symptomatic seizures may occur, it is very rare and should exclude other etiologies such as metabolic disturbance, trauma, and drug abuse (36). Drug-related acute symptomatic seizures may occur following the administration of meperidine, methaqualone, glutarimide, theophylline, isoniazid, imipenem, cefepime, and chlorpromazine (36, 41). Furthermore, breakthrough seizures can develop when drugs, such as barbiturates and benzodiazepines, are discontinued (41). If any of these factors are determined, correction and treatment are essential.

**Table 2:** Electrolyte level abnormalities associated with acute symptomatic seizures.

	Cutoff values most likely associated with acute symptomatic seizure					
Authors	Glucose	Sodium	Calcium	Magnesium	Urea nitrogen	Creatinine
Delanty et al. (38)	<40 mg/dL	<115 mg/dL		<0.8 mg/dL		
Beghi et al. (27)	<36 mg/dL or >450 mg/dL with ketoacidosis	<115 mg/dL	<5.0 mg/dL	<0.8 mg/dL	>100 mg/dL	>10.0 mg/dL
Nardone et al. (37)		<120 mg/dL (acute) <110 mg/dL (chronic) >158–160 mg/dL (acute) >170 mg/dL (chronic)	>12–13.9 mg/dL (acute) ≥14 mg/dL (chronic)	<1 mg/dL		
Karceski et al. (39)	<36–40 mg/dL >400 mg/dL	<115–120 mg/dL >145 mmol/L	<5.0 mg/dL	<0.8 mg/dL		
Gschwind et al. (40)	<36 mg/dL or >450 mg/dL with ketoacidosis	<115 mg/dL	<5 mg/dL	<0.8 mg/dL		
Beleza et al. (41)		<115 mg/dL	<5.0 mg/dL	<0.8 mg/dL		

## 4 Clinical presentation

Poststroke seizures reflect the extent of excessive neuronal discharge and consequent clinical symptomatology. Tonic, clonic, and myoclonic seizures with other semiological findings, such as lip-smacking and motionless staring, may be observed in clinical practice. Nonconvulsive seizures should be confirmed using electroencephalography (EEG).

Clinical signs and symptoms of seizures occur when symptomatic zones of the brain are involved. This may differ from the seizure onset zone or brain lesion, and clinical features may vary according to the seizure propagation pattern. Therefore, even if brain lesions can be accurately localized on brain imaging, semiology may develop in diverse and complex ways when seizure propagation is rapid (43, 44). Furthermore, when seizure semiology and stroke lesions are correlated, the diagnostic accuracy increases significantly through lateralization and localization. Many studies have reported the characteristics of seizure semiology, depending on the location of the brain lesions (Table 3).

**Table 3:** Brain lesion localization with semiology.

Location	Semiology
Frontal lobe	Abdominal aura (44, 45) Altered awareness (46) Akinetic seizure (mesial frontal, inferior frontal gyri) (44) Atonic seizure (44, 46), tonic seizure (44) Automatism (46) Bipedal automatism (mid-part of the frontal lobe) (45) Versive seizure (contralateral frontal eye field, SSMA) (44, 46) Contralateral head and or deviation (fronto-polar, orbito-frontal) (45) Hypermotor seizure Orbital of the mesial frontal (44) Symmetric bilateral, without dystonia (46) Symmetric bilateral, with strong emotionality and vocalization (46) Prominent bilateral tonic posturing (mid part of the frontal lobe) (45) Myoclonic seizure (primary motor cortex, premotor cortex, or SSMA) (46) Prominent leg movement, fencing posture (SMA) (45) Preservation of consciousness during bilateral motor activity (SMA) (44) Bilateral and more widespread somatosensation (SSMA) (44) Epileptic spasm (46) Nocturnal seizures, especially brief, typically with preserved consciousness (46)
Temporal lobe	Atonic seizure (44, 46) Tonic seizure (46) Unilateral dystonic posturing (contralateral TLE) (45, 46) Unilateral tonic seizure (46) Hyperkinetic movements Symmetric bilateral, without dystonia (46) Symmetric bilateral, with strong emotionality and vocalization (46) Epileptic spasm (46) Automatism Limb automatism (46) Oro-alimentary automatism such as lip smacking, sucking, swallowing, and chewing movement (46) Genital automatism (46) Preserved awareness (non-dominant mesial TLE) (46) Ipsilateral automatism with contralateral dystonic posturing (mesial TLE) (46) Auditory illusory aura (temporal neocortex) (45) Postictal nose rubbing or wiping (ipsilateral TLE) (45, 46) Olfactory aura (uncus of temporal lobe) 2 (mesial temporal lobe) (44) Simple auditory hallucination, like buzz or noise (Heschel’s gyrus in the superior temporal gyrus) (44) Fear (amygdala) (45) Initial motionless staring (45) Ictal vomiting (right temporal) (44, 45) Ictal retching (44) Ictal urinary urge (right temporal) (45) Piloerection (left temporal) (45) Ictal spitting or drinking (right temporal) (44–46) Ictal laughing (hypothalamic, mesial temporal, or frontal cingulate) (44, 45) Ictal speech arrest (dominant, usually dominant hemisphere) (45) Ictal speech, verbalization speech (non-dominant seizures) (44–46) Postictal confusion (dominant) (45) Postictal dyslexia (dominant) (45) Postictal dysphasia (dominant) (45) Postictal aphasia (dominant) (44, 46) Postictal nose rubbing or wiping (ipsilateral TLE) (44, 45) Postictal coughing (45) Psychic aura (temporal lobe convexity, posterior temporal lobe with occipital or parietal lobe, and mesial temporal structure) (44)
Parietal lobe	Altered awareness (46) Complex visual hallucinations, visual illusion (parieto-temporal) (39) Hyperkinetic movements Asymmetric, with marked dystonia and vocalization (46)
Occipital lobe	Visual phenomenon (flickering lights, spots, lines, images, and visual field defect) (45) Visual hallucination (45) Elementary visual features, which lack form, color, depth, and movement tend to be foxed on a predictable area of the contralateral visual field (Area 17) (45) More elaborate visual hallucinations, with the features of recognizable form, color, depth, and movement, usually confined to the contralateral half of the visual field (Areas 18 and 19) (45)
Others	
Dominant opercular Insular cortex Insulo-opercular area	Alterations in speech (speech may be typical dysphasic speech) (45) Gustatory aura (44), autonomic alterations such as palpitation, sweating, and goose bumps (44) Preserved awareness (46) Nocturnal hyperkinetic seizure (46)

SMA, supplementary motor area; SSMA, supplementary sensorimotor area; TLE, temporal lobe epilepsy.

### 4.1 Most frequent presentation

Poststroke seizures primarily manifest localization-related seizure semiology, depending on the location of the brain lesion. One-third of all seizures are generalized tonic–clonic seizures (GTCS), whereas two-thirds present as focal seizures, with status epilepticus observed in 9% of cases (47, 48). Focal seizures are common in early seizures, whereas generalized seizures are common in late seizures (47). In patients with ischemic stroke due to large vessel occlusion, seizures occurring within 24 h were predominantly focal seizures or GTCS, whereas seizures with impaired awareness were more common after 24 h (49).

## 5 Assessment and diagnosis of poststroke seizures

### 5.1 History taking

Video-EEG monitoring of all patients with stroke is practically impossible because of time and cost limitations and legislations in different countries. Epileptic seizures are commonly missed by the witness, as most poststroke seizures end within 5 min. Consequently, history taking is the simplest and most vital step in diagnosing poststroke seizures. Although clinicians should carefully listen to patients’ subjective complaints, they should also be familiar with seizure semiology and auras. During interviews, clinicians should ask relevant questions to ensure a correct diagnosis because many patients cannot describe their symptoms concretely and objectively. To ensure an accurate diagnosis, history taking should be divided into preictal, ictal (seizure), and postictal phases. In the preictal phase, determining the various auras of the patient in different environments is important. For example, symptoms appearing while eating, talking, walking, or waking up at night to use the bathroom may be helpful in differential diagnoses. Regarding aura history, confirming the presence of a specific and detailed aura, such as an epigastric rising sensation, hallucinatory taste or smell, ambiguous feelings of fear, anxiety, familiarity, or panorama-like scenes, can help diagnose seizures more accurately than nonspecific symptoms such as headache, dizziness, and nausea (50).

However, the symptoms that develop in the ictal phase cannot be described clearly unless the witness is a clinician. It is therefore crucial to determine the patient’s ability to communicate during a seizure, head version, uneven pupils, patient’s posture, and whether the seizure is tonic or flaccid. Furthermore, abnormal movements, tremulous or myoclonic, such as repetitive, regular muscle contractions, should be determined to ensure accurate diagnosis. In the postictal phase, investigating the presence of confusion, one-sided weakness (Todd’s paresis), or dysphagia is crucial to enable localization or lateralization (50).

### 5.2 Semiology assessment

Although clinicians may witness poststroke seizures directly in some cases, many cases rely on witness statements. Hence, semiological assessment is important for determining epileptic seizures. Moreover, even if a clinician witnesses the seizure, diagnosis may be difficult in cases with nonconvulsive seizures, unusual seizure semiology, or seizure-related sensory symptoms. In such cases, additional tests, such as EEG, magnetic resonance imaging, and laboratory tests, can aid in the diagnosis. In addition, it is necessary to check for an altered mental state, corporeal localization or lateralization, and somatosensory symptoms and autonomic changes and analyze seizure-like activities (51). If the semiology does not indicate a tangible expression of epileptic seizures, another disease should be suspected.

### 5.3 EEG

EEG can aid in the effective evaluation of the brain condition of patients, as it provides real-time and dynamic information on brain function in a simple and noninvasive manner. It effectively distinguishes non-convulsive seizures from autonomic syncope, movement disorders, and pseudo-seizures. EEG has a high sensitivity for the immediate detection of brain ischemia and particularly helpful in cases of large acute infarct volumes (52, 53). In cerebral ischemia, the amplitude and frequency of the EEG waves decrease (54). Acute cerebral infarction may show lateralized or focal slowing patterns on EEG (54). Continuous polymorphic delta with suppressed alpha or beta activity observed in the ischemic hemisphere indicates a poor prognosis (54). Good prognosis is indicated with only delta-to-theta slowing, with no or minor slow activity, and a relatively well-maintained background frequency are observed (54). A recent prospective study investigated whether early (within the initial 72 h) EEG abnormalities could predict poststroke epilepsy during the first year after stroke; the study reported that background asymmetry and interictal epileptiform discharge were independent predictors (55). Another study suggested that there was a 3.2-fold increase in the risk of unprovoked seizures when background asymmetry was observed in the first EEG poststroke and a 3.8-fold increase when interictal epileptiform discharge was observed (55).

Antiseizure medications should be administered when clinical seizures are observed in poststroke patients. However, ASM administration in asymptomatic patients with abnormal EEG findings (e.g., sharp waves or sharply contoured lateralized periodic discharge [LPD]) is debatable in critically ill patients of stroke. The ictal-interictal continuum pattern on EEG can be used as a reference for clinical management, as it suggests possible electrographic seizures (Table 4) (56). A brief, potentially ictal, rhythmic discharge pattern on EEG indicates a seizure onset zone; critically ill patients with this pattern may develop refractory seizures (Table 4) (57). In cases where the EEG pattern satisfies the electrographic seizure or ictal-interictal continuum criteria according to the 2021 American Clinical Neurophysiology Society guidelines, ASM should be considered (Table 4) (56). Furthermore, time-locked LPD is clinically correlated with focal motor jerks, strongly suggesting that epileptic seizures require ASM administration. However, if the discharge frequency does not satisfy the ictal-interictal continuum (<1 Hz) criterion and the shape is less sharply contoured (>200 ms) with LPD, ASM prescription may be suspended. This is due to the possibility of a bystander epiphenomenon, resulting from brain injury without an ongoing insult resulting from a simple structural lesion (58, 59). Generally, one unprovoked seizure with EEG showing prominent epileptiform discharge should be considered an epilepsy based on a seizure recurrence rate ≥ 60% (59, 60). However, applying this in poststroke seizures is difficult because a spike or sharp wave may be observed on EEG due to an epiphenomenon induced by a current acute structural lesion during stroke. In this case, EEG changes induced by the structural lesion (epiphenomenon) disappeared over time when the stroke was properly managed. The 2017 European guidelines do not overlook paroxysmal EEG phenomena as a clinical basis for predicting the recurrence of poststroke seizures (25).

**Table 4:** American Clinical Neurophysiology Society terminologies and definitions for electroencephalography (2021) (56).

Electrographic seizure
Epileptiform discharges or sharply contoured discharges averaging >2.5 Hz for ≥10 s
Any pattern with definite evolution lasting ≥10 s
Brief potentially ictal rhythmic discharges
Focal or generalized rhythmic activity >4 Hz (at least six waves at a regular rate) lasting ≥0.5 to <10 s
Ictal-interictal continuum
Any periodic discharge of spike/sharp wave pattern that averages >1.0 Hz and ≤ 2.5 Hz over 10 s
Any periodic discharge of spike/sharp wave pattern that averages ≥0.5 Hz and 1.0 Hz over 10 s and has a plus modifier^a^ or fluctuation^b^
Any lateralized rhythmic delta activity averaging >1 Hz for at least 10 s with a plus modifier or fluctuation

^a^Plus modifier: An additional feature that renders the pattern more ictal (fast rhythm, rhythmic activity, and spike/sharp waves).

^b^Fluctuation, ≥3 changes not more than 1 min apart in frequency (by at least 0.5 Hz), morphology, or location.

## 6 Differential diagnosis of poststroke seizure

### 6.1 Poststroke alterations in the autonomic system

Patients with stroke may show impaired autonomic function, such as a high incidence of orthostatic hypotension, arrythmias, and syncope, owing to their decreased ability to maintain cerebral blood flow. Although the precise underlying mechanisms remains unknown, several studies have proposed possible mechanisms. For example, damage in ischemic stroke can affect nuclear tractus solitarius signaling, resulting in a constant sympathetic activity that increases the resistance of adrenergic beta receptors to stimulation (30). Meanwhile, other studies have postulated that impaired autonomic function occurs when cerebral perfusion decreases in response to a significant decrease in central blood pressure, potentially due to the withdrawal of excessive sympathetic tone (25). A heart rate variability test was conducted to check for reduced cardiac baroreceptor reflex sensitivity and vagal inhibitory outflow (50). Because autonomic dysfunction symptoms may resemble epileptic seizures, unnecessary ASM use should be avoided in the differential diagnosis.

### 6.2 Movement disorder

Abnormal poststroke movements are not necessarily the manifestations in epileptic seizures. Therefore, understanding semiology is crucial. Poststroke movement disorder (PSMD) is a poststroke abnormal movement that affects 1–4% of all stroke patients (55). A study analyzing 284 published cases revealed that ischemic stroke accounted for 75% of all PSMD cases. Common sites of the stroke lesions included the posterolateral thalamus (23%), putamen (19%), and caudate nucleus (14%) (61). Because approximately 46% of PSMD cases develop within 7 d poststroke, they overlap with early seizures; therefore, differential diagnosis is vital (61). PSMD after an ischemic stroke can appear quickly, usually within 1 month (61, 62). The frequencies of abnormal movements were as follows: dystonia (23%), chorea (16%), and myoclonus (15%) (61, 62). Chorea and myoclonus often occur within 7 d (61, 62). Movement disorders after a hemorrhagic stroke, commonly including dystonia and tremor, appear more frequently after 6 months (61, 62). In PSMD, owing to stroke lesions, the onset time of caudate lesions is relatively long (approximately 6 months). When the lesion is located in the posterolateral thalamus or putamen, PSMD commonly occurs within 7 d (63); therefore, differentiating it from early seizures is important (63). Because 84% of myoclonus cases induced by PSMD improve naturally when appropriately diagnosed, unnecessary use of ASM can be avoided (61).

## 7 Treatment

Primary ASM prophylaxis is not recommended, as it has not been sufficiently proven to reduce acute symptomatic or unprovoked seizures or to improve functional outcomes or mortality (25). Short-term ASM treatment for 1–4 weeks is used for acute symptomatic or early seizures, as the risk of recurrence is generally low (25). European guidelines do not recommend secondary prophylactic ASM for poststroke seizures. Patients experiencing one acute symptomatic seizure within 7 d have a 10–20% chance of experiencing recurrent acute symptomatic seizures; therefore, secondary ASM prophylaxis is not required (25, 64). Despite the relatively low recurrence rate, short-term ASM is used in patients with a pathophysiological background. ASM may decrease neuronal excitotoxicity, peri-infarct depolarization, and inflammatory responses (65). Some studies recommend short-term ASM treatment in early seizures to reduce the tendency of clinical worsening in the acute phase. This approach relies on pathophysiological considerations, including reduced brain perfusion conditions such as stroke with hemodynamically relevant stenosis, brain edema, and vasospasm after subarachnoid hemorrhage (66, 67). However, guidelines recommend tapering of ASM after the acute phase because the low 10-year risk of unprovoked seizure incidence after one poststroke acute symptomatic seizure (30%) (25). The risk of recurrence of unprovoked poststroke seizures within 10 years is high (70%); therefore, secondary ASM prophylaxis is recommended (5, 25). Long-term ASM use is recommended for poststroke unprovoked seizures because the high risk of seizure recurrence when ASM is discontinued (≥50%) (5, 25).

In summary, long-term ASM use is not recommended, except for poststroke unprovoked seizures. However, it can only be used briefly in the acute phase, depending on the patient’s condition, consistent with the definition and treatment strategy for epilepsy provided by the ILAE (8, 41). ASMs, such as lamotrigine, carbamazepine, lacosamide, levetiracetam, phenytoin, and valproate, may be used (68). A multicenter randomized controlled study suggested lamotrigine is more effective as a first-line treatment for patients with focal epilepsy than levetiracetam or zonisamide (69). Regarding poststroke seizure treatment, the efficacies among lamotrigine, levetiracetam, and sustained-release carbamazepine did not differ; however, lamotrigine and levetiracetam were more tolerable than carbamazepine (70, 71).

Administration of an intensive statin dose in patients with stroke reportedly decreases early or late poststroke seizures. Moreover, when statins were administered for ≥2 years, the risk of poststroke epilepsy decreased, regardless of whether statins were administered before or after stroke (72–74). The precise antiseizure mechanism of statins is unknown; however, several theories have been proposed. First, neuroinflammation caused by stroke increases nerve excitability, inducing the secretion of abnormal neurotransmitters by increasing BBB permeability, and leading to seizures by exacerbation of cerebral hypoxia. Statins prevent seizures by exerting anti-inflammatory actions, including regulating blood brain barrier permeability (75), modulating endothelial nitric oxide (76), controlling proinflammatory genes, pro-inflammatory cytokines, and free radicals, and inhibiting lipid peroxidation. While acute ischemia elevates glutamate levels (77, 78), statins inhibit the excitatory toxicity of glutamate by reducing the activity and absorption of N-methyl-d-aspartate receptors and regulating intracellular calcium levels (75, 79, 80). *Bax* induces apoptosis, whereas *Bcl* inhibits apoptosis (19). Statins affect apoptotic pathways associated with these genes and increase neuronal survival, thereby preventing epilepsy (19).

## 8 Conclusion

**Figure 1:**
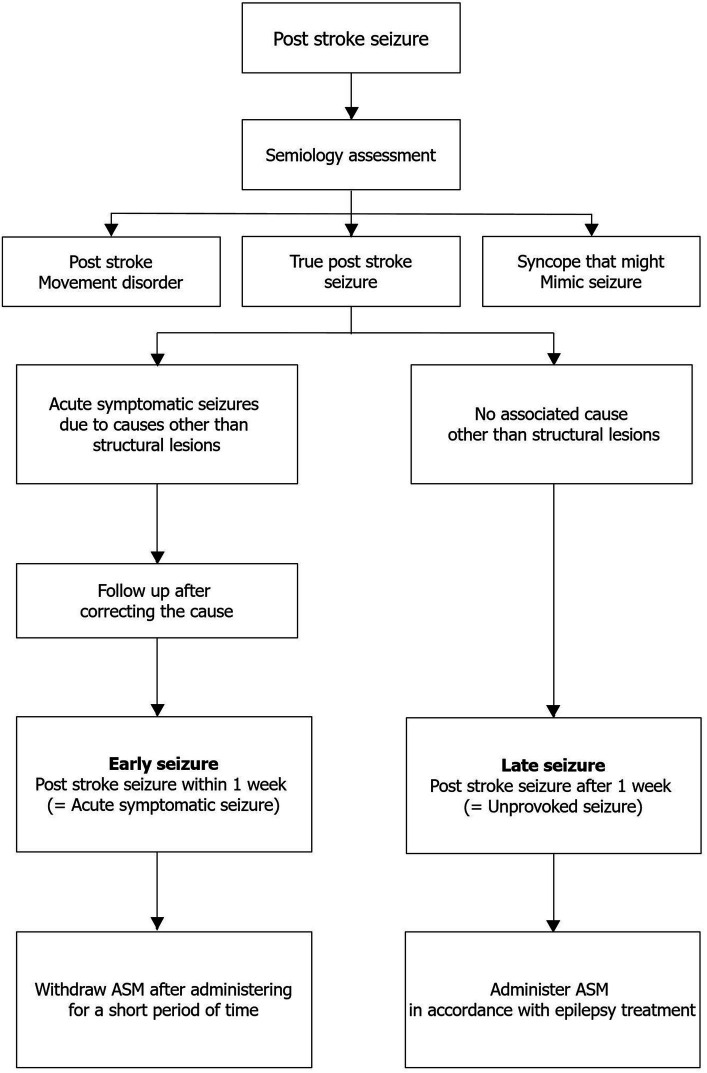
Clinical approach and management of poststroke seizures. ASM, antiseizure medication.

Poststroke seizures are common complications of stroke. It is pivotal to systematically approach, evaluate, classify, and manage them (Figure 1) and differentiate them from abnormal movement disorders, syncope, and psychogenic nonepileptic seizures based on semiology. A systematic approach and identification of factors other than stroke-related structural lesions that can cause acute symptomatic seizures are important (Figure 1). Once a poststroke seizure is confirmed, we can effectively treat patients, improve their prognosis by determining whether it is an early or late seizure, and plan a treatment strategy appropriate for their condition.

## Author contributions

HR: Conceptualization, Formal analysis, Funding acquisition, Investigation, Methodology, Validation, Writing – original draft, Writing – review & editing. HKi: Conceptualization, Data curation, Formal analysis, Investigation, Methodology, Validation, Writing – original draft. B-SS: Data curation, Formal analysis, Investigation, Project administration, Supervision, Writing – review & editing. HKa: Conceptualization, Investigation, Resources, Supervision, Validation, Visualization, Writing – review & editing.
